# Anticipation dialogs in Vermont’s system of mental healthcare: Sustaining the growth of a dialogic practice culture

**DOI:** 10.3389/fpsyg.2023.1084788

**Published:** 2023-03-20

**Authors:** Zelda Alpern, Sarah Binshadler, Ashley Oakley

**Affiliations:** ^1^Counseling Service of Addison County, Middlebury, VT, United States; ^2^Middlebury College, Middlebury, VT, United States

**Keywords:** open dialog, anticipation dialogs, dialogic practice implementation, community mental health, inpatient, person centered rounds, collaborative network approach, dialogic systems change

## Abstract

Open dialog is both a therapeutic approach and a way of organizing the system of mental healthcare that has been evolving in Finland since the 1980s. In Vermont, over the last decade, there has been an organic statewide effort to begin to integrate dialogic principles into the public system of mental healthcare. Because of the organic nature of these initiatives, there have not been coordinated systemic changes to support dialogic practices. To learn what visions participants in dialogically informed practice contexts have for the future as well as what structural innovations would support these visions anticipation dialogs were offered at three dialogically informed community mental health centers and one public psychiatric. The anticipation dialog was developed in Finland during the late 1980s to aid stuck professional and social networks in finding ways to move forward looking back from an imagined positive future. Twenty-seven multidisciplinary staff members and one service recipient participated in the dialogs. The authors conducted a multi-step process of thematic discourse analysis of all 4 anticipation dialogs. Findings underscore dilemmas entailed in growing a dialogic practice system, including the toll systemic uncertainty takes on workers in the system and the simultaneous pull to offer some amount of open-endedness to the system change process in the spirit of inclusiveness, mutual trust, democracy, and reducing hierarchy. Other key findings influencing sustainability of dialogic practices in community mental health include integrating dialogic work into roles rather than adding them to existing responsibilities. Our experiences indicate that anticipation dialogs may be a way of conducting systemic research that contributes to the forward momentum of system innovation. Offering a greater length of time for organizational anticipation dialogs would be valuable, as would centering the voices of clients and their networks.

## 1. Introduction

Open dialog is both a therapeutic approach and a way of organizing the system of care (Seikkula and Olson, 2003, p. 1). As such, successful integration of these practices into services must reckon with the larger systemic context. Any attempt at systems integration of dialogic principles also begs the question of exactly how this can be done *dialogically* as an organic process that staff gravitate toward on their own volition, described by Smith (2022, p. 175) as “collaborative practice development.” To proceed “organically” respects the subjectivity of workers in the system, does not run the risk of workers participating in a dialogic process who are not themselves open to such a process (Parachute NYC, 2015, p. 42, 72) and potentially ensuing negative consequences for clients and their social networks. However, an organic approach that develops in a nonlinear rhizome-like manner (Florence et al., 2020, p. 10), can make it difficult to develop the infrastructure and systems necessary to foster dialogic responses.

It can be argued, that because of the small population and highly democratic culture of Vermont, the social services system is well positioned to support innovation and dialogically informed practice. In spite of this, there are many potential barriers to integration of the principles of open dialog including but not limited to Federal and state funding structures and requirements. This paper briefly describes how inpatient and outpatient staff at four sites that have been training in and practicing dialogic approaches hope to change their systems of care to better align with the seven principles of open dialog. Staff who train in open dialog are faced with the challenge of integrating dialogic practices into varying contexts that are not necessarily set up to support these practices. This “disruptive innovation” (Parachute NYC, 2015, p. 28) can at times be experienced as internal dissonance between the adhering to the principles of dialogic practice and attempting to do so in trainees’ actual work contexts. This dissonance can have a range of impacts on staff morale and the sustainability of dialogic practices in Vermont. We invited staff to share their visions through anticipation dialogs in which actors in stuck systems are able to envision a way forward by looking back from an imagined positive future (Arnkil T., 2019, p. 38). Here, we analyze participants’ visions for change as well as their perceived barriers to systemic integration of dialogic principles in Vermont’s system of care. We go on to offer reflections that may be worth considering for others who hope to integrate dialogic principles into community mental health and hospital settings.

At the time of initiating this project, the authors, social workers at the Counseling Service of Addison County, were concerned that the momentum we had worked hard to achieve in developing dialogic responses to mental health in the state of Vermont were being undermined by the level of crisis in our system of care and our larger society. This crisis was brought about by the pandemic, increasing social and political polarization and a national shortage of human service workers. We were also informed by the idea that times of crises hold within them the possibility for profound and worthwhile change. We believed that inviting staff to participate in anticipation dialogs would offer a way to extricate themselves from this confounding moment and look back on it from an imagined positive future. We hoped that this would offer participants a sense of reprieve, joy, and connection in the moment, as well as some nourishment to help them to continue on this dialogic journey. As Seikkula et al. (2003, p. 197) write, “to enhance commitment, it is necessary to encourage credible hope.” We also hoped that it would increase a sense of direction and accountability. In addition, we hoped we might offer any learnings from this process to the Vermont Department of Mental Health, so that in better understanding our visions and the associated barriers, they might have a clearer sense of how to support our efforts.

### 1.1. Vermont practice context

Based on the estimation of the 2018 US Census, Vermont’s population was 645,570,^1^ the least populous state in the nation and is known as a “human-scale democracy.”^2^ Vermont’s 14 counties are served by 10 designated mental health agencies (DAs) and two specialized service agencies (SSA’s). The DAs have autonomy to design their services so long as they conform to standards set by public and private health insurance, include a 24-h mental health crisis service, community psychiatric support for people who have had frequent and/or lengthy psychiatric hospitalizations, developmental services as well as community and school-based support for youth and families. These agencies are overseen by the Vermont Department of Mental Health. The Department of Mental Health oversees one public Vermont Psychiatric Care Hospital and has administrative ties to five other psychiatric inpatient units within the state.^3^

In 2011, the crisis of Tropical Storm Irene’s flooding of Vermont’s statewide psychiatric hospital gave rise to the state’s willingness to redirect funds toward enhanced community-based responses to mental health crises with the aim of hospital diversion (Smith, 2022, p. 171). Some Vermont-based psychiatric survivors were advocates of the state pursuing open dialog as it prioritizes the agency of the person at the center of concern, increases choice with regard to mental health treatment, and mitigates against coercion (Anonymous, 2019). Some administrators and service providers within the designated mental health agencies (DA’s) and Vermont Department of Mental Health were attracted by the outcomes reported by Western Lapland. According to a study conducted in 2011, using the open dialog approach, as of 2005 new cases of schizophrenia in Western Lapland decreased from 35 cases per 100,000 individuals to two cases per 100,000 individuals (Seikkula et al., 2012). In addition, the DUP (the duration of the untreated period) had declined from 2 to 3 years in Finland’s traditional psychiatric system to 3 weeks in Western Lapland; and 84% of individuals served had returned to full employment. Furthermore, this study had replicated the following results of the period from 1992 to 1997 in which only 35% were treated with neuroleptics, 81% experienced complete remission of symptoms, and 81% had achieved full employment (Seikkula, 2002). A follow-up study documented greatly reduced hospitalizations, use of neuroleptics, and utilization of disability benefits (Seikkula and Arnkil, 2006). In a 19-year follow-up study, Bergström et al. (2018) note that indices of hospital days, use of neuroleptics, and reliance on disability benefits continued to remain lower with people who were responded by practitioners of open dialog. These outcomes were resonant with those of the Vermont Longitudinal Project, the longest study of deinstitutionalization and the second longest study of people diagnosed with schizophrenia which found: 62–68% of “people who were expected to grow old and die at Vermont State Hospital reclaimed their lives,” 81% were able to care for themselves (Harding, 2014).

Through the advocacy efforts of Vermont psychiatric survivors and leaders within several of the DAs, beginning in 2012, funding from the Vermont Department of Mental Health (DMH) was secured to support training in dialogic practice open to workers in the Vermont system of care offered by the Institute for Dialogic Practice. Leaders at the Howard Center, United Counseling Service, Vermont Psychiatric Care Hospital, the Counseling Service of Addison County, and DMH went on to create a statewide training program that would be viable within the context of community mental health with the support of trainers from Tornio, Finland, Norway, Berlin, as well as from Parachute New York and Advocates in Massachusetts. The training was intentionally multidisciplinary; including psychiatrists, case managers, peer support workers, social workers, residential staff, psychologists, nurses, mental health technicians, and mental health counselors working primarily in adult mental health, but in youth and family and developmental services as well. These staffs served both people experiencing first time crises and people who have had long-term involvement with the public psychiatric system. From the beginning, the intention was to inquire into offering dialogic responses to people experiencing a wide range of difficult situations, not solely limited to what has come to be called “early episode psychosis.”

In Vermont community mental health, open dialog-informed practice is referred to as the Collaborative Network Approach (CNA), to underscore that we are not working within a treatment system that is designed to embody the principles of open dialog. While profoundly inspired by the seven principles of open dialog (immediate help, social network perspective, flexibility, responsibility, psychological continuity, tolerating uncertainty, and dialogism), we are operating in different contexts, with differing opportunities and constraints, and must develop approaches that respond to the needs of the particular contexts in which we practice. Although the Vermont Department of Mental Health and the leadership of early adapter mental health agencies and hospitals have been supportive of CNA training and practice, there has not been a comprehensive, systemic commitment to the principles of open dialog or remaking our system of care to be in accordance with these principles.

Some of the participating agencies have also offered in-house training conducted by graduates of the statewide training. At the time of writing this article, we have held four 15-day intensive level 1 statewide trainings and four 10-day level 2 statewide trainings. A total nine agencies have participated including two hospitals and seven community mental health agencies, some of which include residential programs. Of these, three outpatient community mental health centers, Howard Center (HC), United Counseling Services (UCS), Counseling Service of Addison County (CSAC), and one involuntary inpatient facility, Vermont Psychiatric Care Hospital (VPCH), were the initial organizations that came together to plan the statewide training, and who likewise participated in this study. From the outset of this collaboration, each were at different stages of beginning to work with dialogic principles in their own contexts. As we have continued to collaborate on statewide dialogic initiatives, we have each evolved these practices differently in our specific contexts, encountering varied possibilities and barriers.

## 2. Research methods

At the time of this study, the authors were enrolled in *Dialogical approaches in couple and family therapy. Psychotherapy trainers training* organized by Dialogic Partners and the University of Jyväskylä. Included in this program was a 2-day seminar with Tom Arnkil, lead innovator of Anticipation Dialogs. Tom Arnkil also remained available to us for several email, zoom and in-person consultations as questions about methodology arose. We began with the following research questions: What were the visions held by staff at participating organizations with regard to the integration of dialogic practices in our system of care? What were the perceived barriers to realizing this vision? What steps could they imagine taking to reduce these barriers? What actions could Vermont DMH take to further support the CNA initiative throughout the state?

It was important to us that these questions be explored dialogically so that at a time when our professional relational world was under duress, the research process would utilize practices that strengthen networks through listening and deepening understanding (Seikkula et al., 2003). Dialogic approaches to participatory research have been conducted to useful effect elsewhere both within and outside of the social welfare system (Laarni and Aaltonen, 2014; Soggiu et al., 2021). This approach to research actualizes the concept that networks have no centers because each person is the center of their own network (Seikkula et al., 2003). It builds on the:

“incomplete nature of knowledge and the recognition that different participants use different sorts of knowledge. Thus new understanding could be created by including theoretical knowledge and lived experiences” (p. 228).

To be consistent with the practice of open dialog guided as it is by the co-creation of knowledge, rather than a top-down approach to the production and assertion of knowledge, we chose the framework of anticipation dialogs as a way of gathering knowledge from staff positioned in multiple vantage points within Vermont’s mental health treatment systems.

### 2.1. Brief description of the eight principles of anticipation dialogs

Anticipation dialogs are one-time consults offered to clarify complex situations otherwise known as “multi-agency muddles” (Arnkil T. E., 2019) and to collaboratively find a way forward with stakeholders and colleagues. Tom Arnkil and his workgroup began to develop this practice at the Finnish Institute for Health and Welfare in the late 1980s. Continued research into and development of anticipation dialogs were organized by Finland’s National Research and Development Center for Welfare and Health along with several Finnish cities through the 1990s (Seikkula et al., 2003). These initiatives aimed to develop resource-centered methods, a network-oriented work approach, and service structures that transcend sector boundaries to prevent the iatrogenic fragmentation that occurs when a family or child is at the nexus of many specialized professional providers and systems. Akin to open dialog, this innovation privileges respectful and valuing ways of working with clients and their natural supports, and attends to the resilience of clients’ relational and psychological resources (Seikkula et al., 2003).

Anticipation dialogs exist in an imagined positive future and are underpinned by the eight principles highlighted in bold that follow (Seikkula et al., 2003). Two workers from outside the client/provider network offer facilitation to mitigate against the ways that professional hierarchies within agencies and across sectors can act to silence voices, and” to curb cycles of domination and blame” (p.197). In an organizational anticipation dialog, time would be taken to note positions of influence among participants, and to invite those with the least influence to be the first to speak. Beginning with the clients who answer one by one, the facilitators invite those present to imagine a future (the timing of which is agreed on collaboratively at the outset of the meeting) in which the current troubles have been resolved. A future perspective is the basis for coordination, as it offers participants freedom from the stuckness of the moment and makes all equals in the face of no one participant being able to “know the future” and making space for all participants to become curious about one another’s anticipations (p.198).

They ask:

1. *“A year has passed, things are quite well. How are they for you? What are you especially happy about?”*
2. *What did you do to bring about these positive changes? Who helped you and how?*
3. *What made you worried a “year ago” and what lessened your worries?* (Arnkil T., 2019, p. 38).

Each person’s subjectivity is highlighted as they are asked to share their view, aiding in the “transition from objective problems to subjective concerns” (Seikkula et al., 2003, p.198). While each family member is sharing, other family members and providers are listening without interrupting and without the goal of responding directly. In this way, “voices echo in each other” and polyphony is achieved (Arnkil T. E., 2019, p. 593). Movement forward is through each participant being informed by increased understanding of the other made possible through dialogism, not in an attempt to impose one view of the problem on other, and to attempt to change or control them through hierarchical means. This requires, tolerance of uncertainty, as networks are too complex to be controlled, however, increased understanding of one another is possible.

Once it is time for the professionals to share (or in the case of organizational anticipation dialogs, for those of more influence to share), they are in a position of being informed by what they have heard from those who have already spoken, and how this has acted in and on them. To foreground the reciprocal character of professional work, the facilitators invite them to speak subjectively, from the vantage point of their worries, rather than from a monologic vantage point of naming the problem of others in objective terms. The anticipation dialog itself is a series of experiments in thought and action that honors the experimental rather than certain nature of all human activity, helping to level the playing field in the dialog and open space for new directions to emerge (Seikkula et al., 2003).

Once all dialog participants have shared their responses to the three categories of questions written above, the facilitators invite everyone back to the present moment and use the they have taken from the “recalled future” to agree together on who will do what with whom next. While the plan of action is important, its value is based on what occurred during the process of generating it: experiences of listening and being heard engendering all participants to move forward in a relational context of increased understanding and respect for each other’s particular vantage points (Arnkil T., 2019).

### 2.2. Adaptation of anticipation dialogs in this study

We conducted four anticipation dialogs as case studies with four early adapter agencies: Vermont Psychiatric Care Hospital (VPCH), Howard Center (HC), Counseling Service of Addison County (CSAC), and United Counseling Service of Bennington County (UCS). Leaders in CNA at each organization were sent an email outlining the purpose and intention of our research, which was described as: offering an opportunity to share visions of how they would like to see the Collaborative Network Approach (CNA) and/or dialogically informed practices to have taken root in their work context within the next several years as well as with the hopes of keeping CNA’s momentum going, identifying what concrete steps agencies and the state could take to support these initiatives, and to be supported by one another’s visioning processes. We requested that 3–7 staff from each respective agency participate, and that staff be included from different hierarchical levels of influence and varied professional roles. We offered to meet *via* zoom or in person. Two of the three authors of this article were to facilitate the dialogs at UCS, VPCH, and HC. Outside facilitators were engaged to facilitate the anticipation dialog at CSAC. Outside facilitators were engaged to so that authors: (1) might have a firsthand experience of “being in the dialogue” as a way to help us reflect on the process of utilizing anticipation dialogs in this way; (2) could contribute to the development of CSAC’s vision; and (3) to open up more space for CSAC dialog respondents to speak freely.

The first anticipation dialog took place in person at the Vermont Psychiatric Care Hospital on October 20, 2021 and included five participants from the sectors of nursing, psychiatry, psychology, management, and social work. Our second anticipation dialog took place *via* zoom on October 22, 2021 with the United Counseling Service of Bennington County and included four participants occupying different roles in the agency including management, direct service, and clinical staff. The third anticipation dialog we conducted was in person with the Howard Center on November 8, 2021 and included three participants who occupied different roles within psychiatry, management, and clinical work. Our final anticipation dialog took place at CSAC on April 4th and included 16 participants in total, eight in an inner circle and eight in an outer circle. The inner circle consisted of participants spanning clients, peers, clinical staff, psychiatry, and management. This format was slightly different in that the outer circle participants were given an opportunity to reflect from a future position on what they had heard the inner circle participants express on two separate occasions. Unlike dialogic meetings, these reflections were not shared with an opportunity for the inner circle to respond to them, rather they were shared within time constraints with the intention of the inner circle hearing them and then responding to different prompts posed by the facilitators.

As suggested by Arnkil T. (2019), to begin the anticipation dialogs, we collectively imagined the time frame of the dialog to be in the future. This time frame was set by collaboratively asking the participants how far in the future a positive reality in the implementation of dialogically informed practices would be possible. The anticipation dialog questions were then asked in a sequential order, asking one participant each question at a time. Our intention was to begin our round of questions with the person with the least hierarchical influence answering the first question first. The first question asked was, “What are you particularly excited about in relation to dialogically informed practices in the year ‘x’ and what contributed to these changes being possible?” Following the individual responses to this particular question, we asked each participant to elaborate on the actions they took to contribute to these changes being possible, who helped and to share any steps they took that they were secretly proud of. We asked the participants, one at a time, to give voice to their unique perspectives in answering these questions, aiming to end this round of questioning with the person with the most hierarchical influence answering last.

We then asked the next anticipatory dialog question which was, “What were you worried about ‘x’ years ago and what helped lessen your worries?” This question was intended to begin with having the person with the most influence within the workplace hierarchy answer first. Following the individual responses to this question, we asked each respondent how they contributed to reducing the worry including what actions they took and who helped them. In the same manner as the first question, we asked each participant to respond one at a time and aimed to end with the person with the least influence in the hierarchy answering last.

During the responses to the above mentioned questions, notes were shared with the research participants to ensure that we were accurately capturing their utterances. We concluded our anticipation dialog by reviewing the action steps which were mentioned by each participant, clearly outlining who was going to take responsibility, and clarifying an intended time frame as well as who supported each action taken. We also asked when they would meet to discuss these identified next steps. We then asked the research participants how this experience was for them.

We typed up the notes from the anticipation dialogs as well as the action plan and emailed these documents to all of the research participants. In the final portion of our analysis, the authors of this study wrote reflective narratives about our experiences of facilitating these dialogs and shared these with each other.

### 2.3. Anticipation dialog discourse analysis

To analyze the anticipation dialogs, the authors engaged in a multi-step process of thematic discourse analysis (Davies, 2008, p. 186–192). During each anticipation dialog, verbatim transcripts were taken and shared visibly with the participants in real time of their responses to the semi-structured interview questions. These questions aimed to illuminate participants’ hopes, what made this hopeful future possible, their worries, what lessened their worries, and also the steps to be taken by whom and when. We member checked these transcripts by sending them to each participant after they were typed, requesting approval by each participant, and asking for any edits if what was typed did not match what they recall saying. It needs to be noted, not all participants gave feedback to these transcripts.

After soliciting feedback on the transcripts, the authors of this study engaged in a thematic discourse analysis. Salient themes from each anticipation dialog were analyzed and quantified. As much as possible, the data which resulted from the anticipation dialog interviews guided the analysis. No pre-existing theoretic model was used to analyze the data—our analytical process was informed by grounded theory (Glaser and Strauss, 1967). The themes which had the most resonance and frequency across participant organizations, as well as within each organization, became the findings that were generated from this research. This was achieved through an extensive process of reviewing all of the participants’ transcribed utterances, highlighting patterns and then quantifying the frequency of shared themes used by different persons in their responses to the same questions.

The first step of this analytical process was to review the responses to the semi-structured interview questions and identify common themes, paying careful attention to the precise words used by participants and their unique meanings. Once these themes emerged, after reviewing all of the utterances and attending to the specific meaning expressed by each participant, we clustered the participants’ responses into sub-themes. After all of the utterances had been reviewed, analyzed, and placed in a thematic category, we were able to quantify the frequency of each theme. This analytical process led us to our findings which will be discussed below.

### 2.4. Reflections on conducting anticipation dialogs in this study

Anticipation dialogs were organized during regular work hours, on-site at the workplace or in one case during work hours on zoom. The impingement of the workday on the dialogs was felt by participants at times needing to leave early or arrive late or step out for portions of the dialog itself. In one case, during breaks, staff refrained from conversation with one another and instead raced to attend to work responsibilities. Whereas in another workplace, staff took the break time to socialize with one another and the facilitators. Mood and the degree to which staff shortages and the pandemic felt oppressive varied from workplace to workplace. The norms of online meetings seemed to detract from the attentiveness of the listening process, as it was more permissible for folks to attend to other work responsibilities when they were not the one being interviewed, or just to turn off their cameras. This also interfered with being able to stick to the order of interviewing in the order of least to most influence as at times participants “popped off the zoom.” In one workplace, it was the first time key staff had met one another in person, due to the pandemic. At times we felt that the anticipation dialog was adding to the worries of staff, putting pressure on them to talk about actions they would take, when they were already feeling so overburdened by the severe staff shortage. On one occasion, before the sequence of questioning began, an impromptu informal discussion took place in which hopes for person-centered-rounds were described by a participant who held a role of power within the organization. It is possible that this may have yielded influence, unintentionally, on what other participants then shared in the dialog. The authors take responsibility for this and recognize the importance of the structure of anticipation dialogs in opening the conversation by first interviewing the person who has a position with the least organizational influence. We noted that in one case, the presence of a director clearly articulating support for this way of working as well as identifying clear funding streams seemed to further invigorate the visioning process, and seemed to motivate other participants to be more involved to take action. Participants expressed gratitude for this process as well excitement for what was to come. In another case, several key leaders and decision-makers who had intended to participate were unable to due to an emergency. This seemed to have negatively influenced the viability of the dialog and the mood. Compounded with recent staff departures, this group seemed to find it more difficult than the others to participate in the dialog from perspective of a positive imagined future. It is also possible that staff’s protectiveness of each other may have inhibited what it felt possible to share. The stress and pressures expressed during the worries may have foreclosed on a sense of viability to build and enlist one another in a concrete plan. It also may be the case that if a whole day had been offered, a plan might have been arrived at that would have been better able to attend to those worries.

One dialog was conducted by facilitators from outside of Vermont—former community mental health workers themselves who were informed by dialogic practice and had been mentors in open dialog to Vermont practitioners. The dialog included a total of 16 staff with an inner circle of eight actively sharing their delights and worries and making the plan, and an outer circle of eight offered reflections to each other in dyads/triads, and then to the larger group. All but one member of the outer circle shared a reflection with the full group. Two of the researchers participated in the outer circle, and one participated in the inner circle. It was difficult to gauge how and in what ways to use our voices to influence both the content of the dialog and the process with regard to several facilitation decisions that the facilitators directed our way. In one anticipation dialog, two service users (a family member and a person at the center of concern) who have experienced dialogic meetings had planned to participate, one was not able to at the last moment, leaving one to hear the possibly overwhelming nature of staff worries. It would have been helpful to take more time to orient service recipients to the anticipation dialog format ahead of time to increase their sense of safety and clarity about the process.

In the workplace with the most people participating in the dialog, there was a lengthy discussion of in which order to respond and how to determine degrees of influence. This led to other portions of the dialog being more rushed. This took so much time that there was less time to develop the action plan at the end of the dialog and there was no opportunity to debrief. It is possible that when involving such a large group, it would be helpful to have a whole day—with lots of breaks built in (Arnkil, 2022)! And perhaps, as noted by Laarni and Aaltonen (2014, p. 326), in a workplace setting an “iterative series” of dialogs may be called for since “the future is difficult to anticipate, the cyclical paradigm can be used to foster and develop multiple perspectives of the future.”

## 3. Results

Please see the tables listing the most saturated themes for each major line of inquiry of the dialog (Delights of working in 2024—Table 1, What made them possible—Table 2, Worries—Table 3, and What lessened worries—Table 4) following the discussion.

**Table 1:** What are you most delighted about in 2024? (Themes with the highest saturation): Out of a total of 49 themes, these 12 were the most highly saturated.

Frequency	Theme
27	Increased morale (satisfaction, inclusion of all, connectedness, trust, and at east)
25	CNA integrated into job descriptions (manageable workload)
23	Culture change
19	Dialogic system expansion (systems change)
14	Person centered care
13	CNA sustainability
13	Dialogic training/ supervision/ orientation
8	Prioritizing resources of natural supports
6	Increased access for CNA to clients and community
5	Peer support and human rights
5	Working in pairs
5	Inpatient/outpatient continuity

**Table 2:** What made changes possible? (Themes with highest saturation): Out of a total of 32 themes, these 11 were the most highly saturated.

Frequency	Theme
26	Training (inter-agency, intra-agency, and wider community)
13	Funding increases (explored and expanded)
13	Increased implementation
11	Support from key decision-makers/leadership
11	Interested staff have more opportunities to participate
9	Cross silo communication/teamwork
8	Wellness as a collaborative project (staff and greater community)
5	Sustainability
4	5-year development plan
4	One-on-one staff conversations about change
4	Values shift for staff

**Table 3:** What were you worried about in 2022? (Themes with the highest saturation): Out of a total of 40 themes, these 12 were the most highly saturated.

Frequency	Theme
11	Low staff retention
9	Lack of support
9	Influences of other forces at play
8	Over-working and burnout
7	Staff not wanting change
6	Lack of funding
6	Tough times
4	Asking staff to do more
4	Inadequate political and economic power/state support
4	Not having capacity for handling demand for CNA
4	People would not show up
4	Priorities would shift to not be patient-centered

**Table 4:** What lessened your worries? (Themes with the highest saturation): Out of a total of 22 themes, these nine were the most highly saturated.

Frequency	Theme
10	Support from key decision-makers/leadership for system change
9	Alignment of infrastructure and systems with dialogic practice
9	Integration of CNA into job descriptions/roles
7	Accessing new funding sources in the public and private sector
6	Having patience while staying engaged with this model (not letting perfect get in the way of the good and continuing to practice dialogically)
4	Having faith that those exposed to this model would find it meaningful and valuable
4	The spread of passion for this work (energy and revitalization)
4	Showing up for one another
4	Building capacity/sustainability

## 4. Discussion

Here we will offer an analysis of the prevailing themes of the anticipation dialogs, offer reflections on the process of conducting the dialogs, note the limitations of this study and, lastly, share some thoughts about this study’s implications for practice. The theme is followed by the number indicating the frequency with which the theme was mentioned by dialog participants.

### 4.1. Analysis of results

### 4.1.1 Delights of working in 2024

*Increased morale* (27) was the most frequently discussed concept. Increased morale encompasses several unifying topics that include respect, trust, happiness, purpose, momentum as well as inclusion of all staff. This concept can be further understood by the themes that immediately follow such as manageable workload, culture change, and dialogic systems expansion (systems change). Seikkula and Olson (2003) have put forward the idea that open dialog is both a therapeutic approach and a way of organizing the system of care. In Vermont, a statewide training in dialogic practice was offered prior to a reorganization of the system of care in a way that would offer training participants clear routes to practice in accordance with dialogic principles. While in account of Smith (2022, p. 173), “Staff report a stronger emotional connection with colleagues and a feeling of being re-energized by working this way,” these data may indicate that over time staff morale would increase to the extent that dialogic systems change offered dialogically trained staff the context in which such practice would be supported rather than against the grain. For example, if workers’ responsibilities included dialogic practice rather than remained the same, where the expectation that dialogic work be done in addition to pre-existing job responsibilities. This theme, *CNA integrated into job descriptions/manageable workload* emerged as both an overlapping yet distinct theme with a saturation of 25. For example, a status quo in which staff continue to carry a full caseload of individual therapy or case management, and work over-time without pay to co-facilitate network meetings rather than revamping the system of care so that attending to networks is built in programmatically and integrated into job descriptions accordingly. Or psychiatrists primarily seeing clients in the context of network meetings rather than trying to squeeze in a network meeting to a full schedule of meeting with clients individually. Themes that were salient to achieving increased staff morale included: increased pay, increased staff, sustainable workload, working in pairs, and democratic and respectful inclusion of all staff.

Reduction of hierarchy was identified as a factor contributing to staff morale as exemplified by the following utterances: “all disciplines of staff are appreciated for their knowledge” and “trust emerged and can work as equals while in clearly defined roles.” A dilemma that surfaces is how to both move forward in reorganizing the system of care to support and embody dialogic principles and be respectful and inclusive of all staff, some of whom are not in favor of this approach.

The theme of *culture change* [Collaborative Network Approach (CNA) Planning Committee, 2022] included transparency, dialogism, increased polyphony, and equality among staff, and a sense that change has been an organic process achieved by modeling and the accumulation of positive outcomes. While the theme of dialogic systems change overlaps with the theme of culture change, we see a difference between the two, where the first points to explicit decisions made to reconfigure the system of care, culture change embodies a more organic change process premised on influence and attraction rather than implementation. Expressions of *culture change include:* “Once staff noticed how beneficial it was everyone *wanted* to do it,” “staff who are practicing show such integrity in what they say and do others are following,” “values have leaked into youth and family,” “there is *a* noticeable shift in how we are having conversations-nothing about me without me,” “staff-wide acceptance of meaning of what patients are saying” and “day to day they [those engaged in services] feel responded to in a way that is rooted in dialogic work,” and “OD is the first thing thought *of* as safety net, way of working, family of choice.” One utterance that was particularly radical imagined a shift to the extent that staff no longer used texts or emails to communicate about people who were engaging in services—please let it be so!

*Dialogic system expansion/systems change* (Rosen and Stoklosa, 2016) was expressed as having achieved a high degree of responsiveness—same day access in outpatient settings, and CNA becoming the primary and initial way of responding to requests for service: “CNA is the modality that is mainstreamed for how to move forward (in every department).” In some cases, CNA was imagined as a specific team of dialogic facilitators (“established CNA team to facilitate meetings that respond immediately, mobile, offer psychological continuity”), while in other utterances, it was imagined that all staff had been trained and “CNA [is] part of everyday workday.” Other utterances describing a vision of dialogic practice included: “We have really slowed down,” “before the intake we are thinking about their community and network,” and “[we have] fully embraced ‘nothing about me without me.” Being able to offer *person centered care* (Davies, 2008) was also high in frequency. This indicates the degree to which dialogic training touches on deeply held humanitarian values for training participants, and how morale is imagined to increase when being able to practice in alignment with these values. In outpatient settings, this was expressed as “power with instead of power over” network participants, and as network participants helping to shape the system change through co-research (Anderson, 1997). An utterance from CSAC included in this theme, “psychiatry as a small part of service not a driver of practice” exemplifies a shift from privileging the agency of the professionals to the agency of the person at the center of concern. The hope was also expressed that psychiatrists could participate in the dialog as a “human being” and not solely viewed through a psychiatric lens. At VPCH, the one inpatient setting, person centered care was primarily defined as offering “person-centered-rounds”—as distinct from rounds where the treatment decisions related to the person at the center of concern are discussed without their presence (Rosen and Stoklosa, 2016).

*CNA Sustainability* (Arnkil T. E., 2019) is related and yet distinct from issues of morale and workload. This theme is largely concerned with workers’ experience of whether the practices of CNA will survive in their work contexts. Factors put forward to help staff be “less worried and more confident, [and have a] sense of ease that dialogic practice will be supported and working” included having many more staff involved so “we will not have to worry what happens if we lose two members of the team,” and managers valuing dialogic work as evidenced by allocating time, resources and funding for it,” to the point that CNA is stable enough so that leadership change (if they do not know about dialogic practice) will not threaten CNA.”

Participants at all sites spoke to the theme of *training and supervision* (Arnkil T. E., 2019), the aims of which included: increasing the number of staff prepared to facilitate dialogic network meetings, familiarizing all staff with dialogic values and principles, embodying dialogic principles in varying contexts—such as residential settings, and increasing community awareness of these approaches. Training included both new staff orientation, community education, in-house training programs and setting aside 30 min of weekly staff meetings to do mini-trainings. Participants also spoke of offering on-going training that was accessible “at good times in their careers when they can also do the work”—further underscoring the link between training and the conditions in which it is possible to practice learnings from the training.

*Prioritizing resources of natural supports* (Seikkula et al., 2012) was also emphasized across all four sites, invoking a paradigm shift from individual-based to network-based engagement—it is the “norm for the network & the person at the center of concern to be engaged.” Participants from VPCH envisioned person-centered rounds in which “natural supports and external providers participate in weekly network meetings.” In an outpatient setting, a staff-person shared, “Before the intake we are thinking about their community and network.” Rather than holding up medication as a solution, one community-based psychiatrist proposed that from the outset we “hear from the whole network and think together about what resources they already have and what we can offer.” This theme encompassed both clients’ pre-existing natural supports, and an orientation toward helping potentially isolated clients to foster new relationships in the community.

*Increased access to CNA for clients/community* (Seikkula et al., 2003) is closely linked to the system change theme and is highlighted because this aspect of system change was mentioned across outpatient settings. CNA was also envisioned as a way to respond to communities in conflict with one another. *Peer support and human rights* (Arnkil T., 2019) is also closely linked to systems change—and was spoken of in conjunction with the advocacy necessary to reconfigure agency leadership structures to include peer support workers, to hire individuals currently engaging in services at the agency, and to increase dedicated hours for peer support staff to facilitate network meetings. This theme is also inclusive of a systematized effort to outreach to people who have experienced involuntary hospitalization to offer network meetings with the aim of restoring trust, validating trauma sustained in the process for all parties involved and gathering learnings in the service of preventing future involuntary hospitalizations.

*Working in pairs* (Arnkil T., 2019), is linked to system change, staff morale, and sustainability. Working in pairs enables the reflecting process, tolerating uncertainty, flexibility, and responsibility. In addition, “people do not feel alone in their work” and can further deepen collaborative, trusting relationships (if all goes well!). *Inpatient/outpatient continuity* (Arnkil T., 2019) was expressed by both inpatient and community-based dialog participants with the idea that the outpatient team would be able to stay connected with the network during hospital stays.

#### 4.1.2. Worries

When the participating organizations were asked what they were worried about, *lack of support from key decision-makers and/or leadership* (Seikkula, 2002) to support systems change was a key theme. “It could fall apart because it’s supported by a small group of people” speaks to a sense of precariousness. Many participants expressed concern about losing momentum in this way of working in connection to lack of support and/or *low staff retention* (Bergström et al., 2018)—increased difficulty filling vacant positions as well as dialogically trained staff leaving positions. One respondent was concerned that “staff would not have the time, energy and passion necessary to make change” another shared, “People are so stressed and tired.”

While these worries were frequently acknowledged, there was also a focus on *not wanting to risk overworking* (Seikkula et al., 2012) remaining staff by *piling more onto their existing workload* (Florence et al., 2020). “We can train people in OD but then they are running on fumes and that will run out without structural change.” Another often-voiced worry, was the *lack of consensus among staff* within agencies about aspiring to the principles of open dialog (Anonymous, 2019). Utterances along these lines included, “other staff would not agree to change [and] if they did not, neither would the system” and worry that the “influence of those who did not want to make changes [who held] priorities that were not patient-centered.” This last worry has implications both for worries about the practice itself, the wear and tear of paradigm differences among staff and the extent to which being in a holding pattern with regard to a more wholesale systemic commitment to these practices decreases staff morale and resilience. It is worth noting that these dialogs were held during the pandemic and a time of increasing political and social polarization and its toll for many was felt both personally and professionally for respondents—*tough times* (Seikkula et al., 2003) gives additional context to worries about overworking staff, low staff recruitment and retention, and concerns about the deleterious effect of an ongoing lack of staff consensus.

#### 4.1.3. What made change possible

When the participating organizations were asked what made changes possible, *offering and expanding dialogic training* (26), both within organizations and among the community, was the theme expressed across organizations with the highest saturation. This is related to other prominent subthemes, primarily the need for additional funding as well as increased implementation of dialogic practice in a context in which this practice is supported holistically. Dialogic practice in a context which is supported holistically translates to offering training and supervision to interested staff, integrating CNA into job descriptions so dialogic work is integrated rather than additive, and increasing collaborative teaming opportunities—for all staff, including psychiatry—to work in alignment with dialogic principles. The second most salient theme, *increased funding* (Arnkil T. E., 2019), was expressed as the need for higher pay, which was also connected to the frequent highlighting of the need for increased staff recruitment and retention. However, *increased funding* was also expressed as greater investment in training, investment, and financial support in novel ways of working, private insurance revenue paying for new CNA-specific positions, and Medicaid and private insurance reimbursement for dialogic practices. *Increased implementation* (Arnkil T. E., 2019) was expressed as hiring more staff with CNA-specific responsibilities, offering training and creating infrastructure to respond immediately, adoption of inpatient person-centered round, and adoption of dialogic intake program, moving toward being able to offer services without imposing a psychiatric diagnosis. *Support from key decision-makers/leadership* (Bergström et al., 2018) included utterances such as “division directors buy in,” support from the state legislature, support from supervisors, leadership “talking about it until people relented.” *Interested staff have more opportunities to participate* (Bergström et al., 2018) was expressed as opportunities for dialogic training and supervision, integration into work responsibilities, and psychiatrists and peer support workers being able to participate as meeting facilitators rather than their roles eclipsing their ability to be seen as facilitators. *Cross silo communication/teamwork* (Seikkula, 2002) was expressed as inter and intra-agency collaboration as well as cross-department, cross-agency, multidisciplinary trainings as a way of breaking down silos. Wellness as a collaborative project (Seikkula et al., 2012) was described as shared by staff for staff, as well as a joint project of the staff and the greater community, for example, having barbeques and engaging in “activities and events together with art and music.”

#### 4.1.4. What lessened worries

*Support from key decision-makers/leadership for system change* (Seikkula and Arnkil, 2006) would be necessary to make such integration possible. For example, “leadership supporting and allowing time to train and work with families.” Participants expressed that *alignment of infrastructure and systems with dialogic practice* (Seikkula, 2002) and *integration of CNA into job descriptions* (Seikkula, 2002) lessened their worries. Such integration would be the corrective to overworking, working “against the grain” of the system, and tolerating the uncertainty of lack of clarity about the direction in which the organization is going; rather, participants envisioned practicing in a systemic context that was organized to support dialogic principles. *Having patience while staying engaged with this model* (Seikkula et al., 2003) speaks to the organic nature of dialogic processes—of tolerating the uncertainty of becoming. One respondent offered, “Faith that once enough people were exposed they would find it valuable and meaningful.” Another recalled, “There was a tipping point and the fire spread on its own.”

### 4.2. Limitations

A significant limitation of this study is that those of us who facilitated the anticipation dialogs have not undergone the 18-day standard of training in that way of working. This may have negatively impacted our ability to conduct the dialogs, our collective understanding of outcomes and explained some of our difficulties with pacing. Frequently, the authors hoped to have more time to engage in a reflective, dialogic, conversation with participants and to allow the participants to reflect on what one another shared. Timing was compromised at times due to accommodating arrival times and the need for participants to exit the dialog.

These dialogs primarily involved staff and did not significantly enlist those with direct experiencing of “being responded to” by our system of care. On the one hand, given the state of duress workers in our system of care were in at this time, it may have been harder for staff to have given voice to this aspect of the visioning process if more service users had been present, for fear that it was not appropriate to talk about the cost of working in the system of care. It may also be true, that if more service users had been present, this would have reinvigorated staff’s commitment to dialogic practice, and informed how best to realize it based on the priorities expressed by service users. Perhaps, letting service users in on the worries held by workers in the system may have allowed more potential ways forward to emerge. Directions for future research might benefit from an anticipation dialog research project informed by the work of Soggiu et al. (2021) who describe a dialogic research process that privileges the role and voices of service users throughout.

There are many overlapping relationships and dual roles in the state of Vermont, for instance, two participants from HC involvement in the CNA statewide planning group with one author of this research. Social and familial relationships exist across agencies and within the community. Often, this interconnectivity is seen as a strength; however; it may have influenced the way participants responded to one another in having deeper awareness of the potential impact of their responses on their colleagues.

All three of the researchers work or have worked at CSAC and were either in the inner or outer circle of the AD as participants. All three of the researchers have been involved in the statewide training as trainees and either trainer or trainer in training. One author of this research was in a workplace supervisory role of the two other authors for the majority of the duration of the research project.

We conducted anticipation dialogs with each organization separately and so each organization was unable to hear and/or respond to the utterances of one another. The context of when these dialogs took place is important to address in that many of the organizations were in the midst of an extreme staffing crisis. The number of participants from each organization was not equal which impacted the representation of utterances unevenly throughout the data. The Counseling Service of Addison County in particular had more participants.

The COVID-19 pandemic, increasing economic distress, and social/political polarization were an ever present influence that deeply impacted working practices at all agencies involved in this study. How these contextual factors were brought to bear on the visions, hopes, and worries expressed within the anticipation dialogs can only be speculated upon. However, it is notable that *increased morale* was the most saturated among delights (27). The specter of these larger world events and socio-political currents have also acted upon the authors of this study, influencing our own morale and the lens through which we have interpreted the data and experienced the dialogs.

It could be argued that rather than being at an impasse, our system—and those of us working in it—was/were in a state of crisis at the time of this research, and that it is open dialogs rather than anticipation dialogs that are called for in moments of crisis (Eriksson and Arnkil, 2009).

### 4.3. Implications for practice

In their study of the evolution of CNA, Florence et al. (2020, p. 688) note, “The combination of working from the ground up, determining how to incorporate network meetings within agencies and having support from the system more broadly were described by participants as key features of the Vermont experience.” These dialogs demonstrate a vision for change in Vermont’s system/s of care to increase the extent to which dialogic work is the norm rather than the exception in how we respond to people seeking support. Each setting has its own particular vision and path in this regard. However, across site, the following common themes emerged as far as what would make change possible: clarity and more decisiveness from agency leadership and DMH in systemically committing to dialogic principles, integrating CNA into workers’ roles and duties, staff recruitment and retention, reducing hierarchy and continued training.

These are noticeably interdependent. For training to be a worthwhile investment, there must be low-turnover. To retain staff and increase morale, it is important to have a clear vision, room for staff to co-create the vision, and have workloads that are reasonable and purposeful. To recruit and retain staff engaged in dialogic work, they must make a live-able wage which requires a systemic commitment from DMH, agency leadership, and the state and national legislature. The human service staffing shortage is a complex issue and not one that has easy answers.

While anticipation dialog participants shared that dialogic practice offers an increased sense of meaning and joy in working together, this in itself needs to be complimented by additional factors to retain staff. Staff retention is pivotal in the sense that if agencies invest in training staff they remain in the public sector. Training needs to be offered in connection to a CNA-specific role in which the principles taught in training can be utilized and supported. Taking on dialogic work in addition to one’s job description risks leading to burnout and reduces the sustainability and growth of these practices. Making dialogic practice an explicit part of someone’s role and not an additive, evolving a systemic context that supports these roles are key to sustaining staff who have come to embrace dialogic work. These themes are connected to the expressed hope for system change and the necessary financing of such change. Funding would need to be expanded and reallocated to support CNA-specific roles, expanded training opportunities, and staff pay increases.

Reducing hierarchy and increasing democracy, collaboration, and inclusion was often expressed as a vision for the future. This again highlights the dilemma of how leadership can be more proactive and decisive about making changes in the system of care, while simultaneously fostering a less hierarchical and more democratic workplace. The dialogic principles have the potential to anchor us, yet a focus on them may also call attention to divergent values among staff thereby heightening tensions in the workplace (Florence et al., 2020)—especially if in order to participate in dialogic work, staff need to do so above and beyond their explicit work responsibilities. Relatedly, staff across the agencies pointed to the significance of staff morale, staff inclusivity, the joy of working together, being part of a meaningful community in which there is mutual respect and interconnection. The high saturation of these themes points to how sensitive staff are to each other. People working together are often sensitive to how connected or disconnected they feel to one another and being in conflict can be difficult. These principles and this way of working therefore can influence morale for better or for worse. This also raises the question of whether tensions experienced between staff with differing relationships to dialogic practice are exacerbated by prolonged uncertainty about whether or not a workplace is shifting toward a dialogic framework, without clear signals and plans from leadership.

As mentioned, dialog participants frequently voiced the hope for system change in a strategic and coordinated way. It is possible that dialogic practitioners having a more regular audience with the department of mental health would improve communication and clarity about making systemic changes, and asking more directly for DMH’s support in creating funding mechanisms and other adaptations so that DMH’s substantial investment in training is more efficiently channeled into practice, and so that staff are not in the dissonant position of learning a new practice that can be experienced as being a square peg in a round role. This may be easier said than done, for as Florence et al. (2021) point out, it may be “better to start somewhere and gradually take up other elements that can be harder to integrate in a system that operates in an antithetic way” to dialogic principles.

As far as we are aware, this is the first time a study has been conducted on the experience of integrating dialogic principles in a multi-agency system of care utilizing anticipation dialogs. When we decided to offer these dialogs, it was with the intention that they might increase a sense of hopefulness, momentum and energy during a chaotic and strained time. We invoked the idea of crisis as opportunity—and we were in the thick of it. There is a feeling now of being able to see the light at the end of the tunnel. In January of 2022, the United Counseling Service launched the dialogic rapid access intake project hinted at in the anticipation dialog of 2021. In September 2022, the Vermont Psychiatric Hospital adopted person-centered-rounds envisioned in October 2021 on one of their units. CSAC launched a dialogic rapid access intake program inspired by UCS in October 2022 and plans launch a hospital diversion program integrating open dialog and intentional peer support in early 2023. The Howard Center has created a new position entitled the Coordinator of Peer Support and the Collaborative Network Approach. The Vermont Department of Mental Health, and CNA leaders a participating agency are in the process of creating a workgroup to identify and attend to barriers to realizing dialogic practice within the statewide system of care [Collaborative Network Approach (CNA) Planning Committee, 2022]. While we cannot presume that the anticipation dialogs that played a role in these developments, it is worth noting. As such, anticipation dialogs may be a way of conducting systemic research that contributes to the forward momentum of system innovation.

It is hard to keep from wondering what these dialogs might have yielded if they had been conducted prior to the outset of the pandemic and the acceleration of a staffing crisis in our system of care. Concerns about sustainability of CNA practices are in a larger context of staff uncertainty about the integrity and longevity of our public system of care as a whole. Rather than arriving at a comprehensive list of actionable items of how we might advance the particulars of dialogic practice, we find ourselves needing to address the more global issue of finding and retaining staff. That said, even prior to the pandemic, staff have spoken to the difficulty of being in a drawn out holding pattern in which, once having become energized and inspired by the dialogic training process, they attempt to practice in ways that increase their workload without a clear commitment from leadership to make systemic changes in accordance with these principles. While at the beginning of Vermont’s inquiring into and gaining experience with dialogic work holding, some uncertainty about how this would be borne out was tolerable, and perhaps necessary as a way to protect a needs adapted rather than a more standardized approach to open dialog, as the years go on, the uncertainty may be at a cost to sustainability. How can decisions be made regarding the advancement of dialogic practice in Vermont in a way that is in keeping with being inclusive, democratic and non-hierarchical? The seven principles of open dialog (immediate response, responsibility, flexibility, psychological continuity, a network perspective, dialogism, and tolerating uncertainty) are interdependent in their work to support a network’s journey through a transitional and chaotic period. In both therapeutic and organizational change processes, a balance is needed between the open-endedness of possibilities, and the safety and stability of a team taking on their share of the responsibility for holding the process (Lennon et al., 2022). We are left encouraged by participant motivation to continue on this path with hopes that this paper may contribute to striking a sustainable balance.

## Data availability statement

The raw data supporting the conclusions of this article will be made available by the corresponding author (ZA), without undue reservation.

## Ethics statement

Ethical review and approval was not required for the study on human participants in accordance with the local legislation and institutional requirements. The patients/participants provided their written informed consent to participate in this study.

## Author contributions

ZA, SB, and AO contributed to the conception, design, data collection, and data analysis and contributed jointly to the initial write-up of this study submitted as a paper required to graduate from Dialogical Approaches in Couple and Family Therapy Psychotherapy Trainers Training sponsored by the University of Jyväskylä. The manuscript for this article was primarily drafted by ZA drawing a great deal from this paper. All authors contributed to the article and approved the submitted version.

## Funding

As stated, all of us worked at the Counseling Service of Addison County for the majority of the duration of this study. However, anticipation dialogs conducted by ZA, SB, and AO, as well as the analysis of the dialogs occurred outside of paid work hours. One author did use 1 day of paid educational leave to complete edits on this article.

## Conflict of interest

At the time this research was conducted, ZA, SB, and AO worked at the Counseling Service of Addison County (Vermont, United States), one of the sites included in the study. One of the authors (ZA) is also the coordinator of Vermont’s Collaborative Network Approach (CNA) State-wide Planning Committee. ZA, SB, and AO have participated as trainees and trainers in the CNA State-wide Training.

The authors declare that the research was conducted in the absence of any commercial or financial relationships that could be construed as a potential conflict of interest.

## Publisher’s note

All claims expressed in this article are solely those of the authors and do not necessarily represent those of their affiliated organizations, or those of the publisher, the editors and the reviewers. Any product that may be evaluated in this article, or claim that may be made by its manufacturer, is not guaranteed or endorsed by the publisher.

## Footnotes

^1^United States Census Bureau (2018). *QuickFacts: Vermont*. [online] Census Bureau QuickFacts. Available at: https://www.census.gov/quickfacts/VT.

^2^Bartlett, E. (2017). "Human-Scale Democracy" Credited for Vermont’s Culture. U.S. News & World Report. [Online]. Available at: "Human-Scale Democracy" Credited for Vermont’s Culture (Accessed September, 2022).

^3^mentalhealth.vermont.gov. (n.d.). Designated Hospitals | Department of Mental Health. [online] Available at: https://mentalhealth.vermont.gov/individuals-and-families/designated-hospitals (Accessed September 25, 2022).
